# Specific Monitoring the DNA Helicase Function via Anchor‐Embedded DNA Probe

**DOI:** 10.1002/advs.202413368

**Published:** 2024-12-31

**Authors:** Keni Ning, Xiaoyan Tang, Zhe Li, Liting Zhong, Yingchen Zhou, Jiaen Wang, Wanyi Huang, Han Zhang, Jiajun Ke, Tiangang Luan, Shuo‐Bin Chen, Junqiu Zhai

**Affiliations:** ^1^ School of Pharmaceutical Sciences Guangzhou University of Chinese Medicine Guangzhou 510006 China; ^2^ School of Pharmaceutical Sciences, Guangdong Provincial Key Laboratory of New Drug Design and Evaluation Sun Yat‐Sen University Guangzhou 510006 China; ^3^ School of Environmental and Chemical Engineering Wuyi University Jiangmen 529020 China; ^4^ School of Life Sciences Sun Yat‐Sen University Guangzhou 510275 China

**Keywords:** DNA helicases, DNA probe, intracellular monitoring, Werner syndrome helicase

## Abstract

DNA helicases play a pivotal role in maintaining genome integrity by unwinding the DNA double helix and are often considered promising targets for drug development. However, assessing specific DNA helicase activity in living cells remains challenging. Herein, the first anchor‐embedded duplex (ATED) probe, **17GC**, is constructed to uniquely monitor the unwinding activity of Werner syndrome helicase (WRN), a clinical anticancer target. This probe integrates biophysical screening and molecular simulation approaches. The **17GC** probe consists of two components: the first one is a bubble structure as an anchor for recruiting WRN in cells, and the second one is GC‐rich double helices on both ends of the bubble, which allow high sensitivity in detecting WRN activity. In vitro evaluations demonstrate that **17GC** is highly sensitive and specific to WRN (LOD = 33.5 pm) compared to a wide range of other enzymes, including helicases and nucleases. Cellular evaluation reveals that the ATED probe exhibits remarkable performance in monitoring WRN helicase activity and assessing the inhibitory efficiency of clinical WRN inhibitors in various cell types. This study introduces a novel approach for designing specific and sensitive probes for DNA helicases in cells, which holds promise for biological characterization and drug development.

## Graphical abstract

An anchor‐embedded duplex (ATED) probe for sensitive and specific monitoring Werner syndrome helicase (WRN) activity and evaluation of helicase inhibitors in living cells.

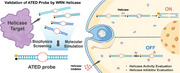

## 1 Introduction

DNA helicases are a class of essential enzymes that play a pivotal role in all organisms by unwinding structured nucleic acids.^[^ ^1^ ^]^ These enzymes are integral to DNA repair systems, which are crucial for maintaining genome integrity, facilitating efficient DNA replication, and enabling tolerance to DNA damage.^[^ ^1^, ^2^ ^]^ The upregulation of certain DNA helicases has been linked to cancerous cells, where they contribute to the proliferation of cancer cells and their resistance to chemotherapy‐induced DNA damage.^[^ ^2^, ^3^ ^]^ Consequently, DNA helicases have garnered substantial attention as promising targets for innovative anticancer therapies.^[^ ^4^ ^]^ To advance the understanding of DNA helicases, there is an urgent need for sensitive and real‐time techniques that can visualize their activity within living cells. Such approaches would help unravel the dynamic roles of helicases and assist in identifying potent inhibitors, thereby contributing to the development of helicase‐targeted therapies. Furthermore, investigating the helicases that unwind specific DNA structures holds the potential to open new avenues for drug discovery targeting cancer and other diseases.

Werner syndrome helicase (WRN) is a RecQ family member often referred to as the “guardian of the genome” due to its ability to unwind a wide range of DNA structures produced during physiological metabolic processes.^[^ ^5^ ^]^ Numerous investigations have consistently demonstrated that WRN is overexpressed in a variety of cancer cells and the activity of WRN is crucial for the survival of certain cancers with DNA repair defects, especially microsatellite instability (MSI) cancer.^[^ ^6^ ^]^ Notably, high‐throughput screening (HTS) has led to the discovery of two WRN inhibitors, which have now entered clinical trials.^[^ ^7^ ^]^ Despite this progress, the development of WRN inhibitors remains limited, largely because of the challenges associated with assessing their unwinding activity.^[^ ^8^ ^]^ One of the primary obstacles in WRN inhibitor development is the significant difference in WRN unwinding mechanisms between extracellular and intracellular environments.^[^ ^9^ ^]^ For instance, WRN interacts with numerous proteins involved in DNA metabolism, and the replication protein (RPA) has been shown to strongly stimulate the helicase activity of WRN.^[^ ^10^ ^]^ These interactions make it difficult to simulate WRN behavior using cell‐free activity assays. Therefore, analyzing intracellular WRN activity is critical for understanding its physiological roles, therapeutic potential, and involvement in cellular signaling pathways.

To date, the most commonly used methods for detecting DNA helicase unwinding activity include electrophoretic mobility shift assay,^[^ ^11^ ^]^ fluorescent analysis based on fluorescence resonance energy transfer,^[^ ^11^, ^12^ ^]^ enzyme‐linked immunosorbent assay,^[^ ^13^ ^]^ magnetic tweezers,^[^ ^14^ ^]^ optical tweezers,^[^ ^15^ ^]^ laminar flow and DNA curtains.^[^ ^16^ ^]^ Nevertheless, these approaches are rarely applied to monitor helicase activity within living cells. In recent years, nucleic acid fluorescence probes have emerged as a promising tool for monitoring the activity of various intracellular nucleases,^[^ ^17^ ^]^ including human apurinic/apyrimidinic endonuclease 1,^[^ ^18^ ^]^ DNA glycosylase,^[^ ^19^ ^]^ telomerase,^[^ ^18a^ ^]^ and O^6^ methylguanine DNA methyltransferase.^[^ ^20^ ^]^ For example, Li group^[^ ^18a^ ^]^ constructed a DNA fluorescent probe whose conformational changes can be triggered by TE‐induced DNA elongation and APE1‐mediated specific cleavage, generating fluorescence signals to image the activity of the two nucleases in an AND‐gated manner. Therefore, employing DNA fluorescent probes presents a promising approach for developing a detection method to assess intracellular WRN activity. Designing and screening a DNA substrate with both sensitivity and selectivity to WRN is crucial in employing a nucleic acid fluorescent probe for intracellular WRN analysis.

**Figure 1:**
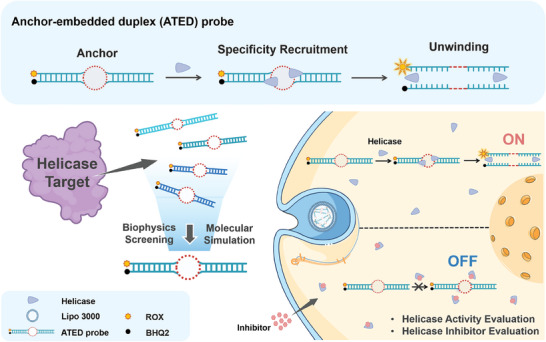
Schematic diagram for the fabrication of ATED DNA probe and intracellular imaging of WRN.

In this study, we designed and constructed the first anchor‐embedded duplex (ATED) probe, **17GC**, for the unique cellular monitoring unwinding activity of WRN. The ATED probe integrates biophysics screening and molecular simulation approaches. By investigating the effect of centrally located bubble structure size, double helix length, and abundance of GC, a sensitive ATED probe that can selectively respond to WRN was developed. This probe exhibited effective differentiation between WRN and nonspecific helicases. Furthermore, the ATED probe was successfully employed to monitor and visualize WRN activity and evaluate the efficiency of various WRN‐targeting small molecules in different living cells (**Figure** **1**). To the best of our knowledge, this is the first study to visualize the unwinding activity of WRN in living cell. This work establishes a foundation for the development of intracellular detection techniques for DNA helicases, represented by WRN, and opens new paths for biological characterization and drug discovery.

## 2 Results

### 2.1 Initial Design of the ATED Probes for WRN

**Figure 2:**
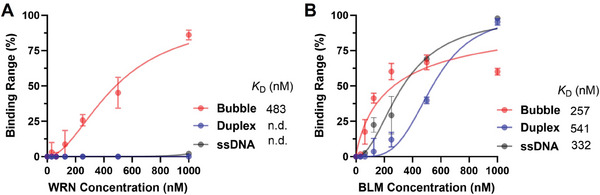
The binding affinity of WRN (A) and BLM (B) to single‐strand DNA, bubble DNA, and duplex DNA was evaluated by filter‐binding assay. Biological replicates (n = 3) were taken. The data are presented as mean ± SEM.

Previous studies have revealed that WRN exhibits higher unwinding activity to bubble DNA than the other RecQ helicases.^[^ ^5^, ^21^ ^]^ This finding suggested that WRN might have a preference for binding bubble DNA over other DNA substrates. Thus, we first evaluated the binding potential of WRN to bubble DNA and figured out whether the bubble could be used as an anchor in the ATED probe for WRN. As shown in **Figure** **2A**, WRN displayed a much stronger binding affinity for bubble DNA compared to duplex or single‐stranded DNA. In contrast, its homologous protein BLM exhibited similar binding affinities for single‐stranded, bubble, and duplex DNA (Figure 2B). These findings inspired that WRN uniquely prefers bubble DNA, which can be leveraged as an anchor for the ATED probe to specifically recruit WRN.

**Figure 3:**
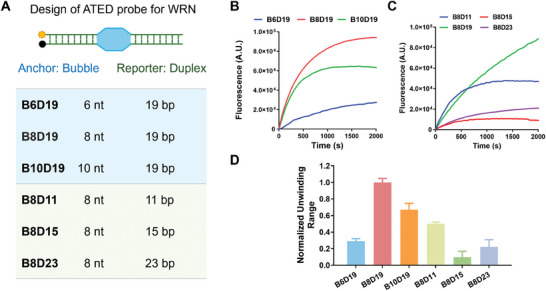
Design and evaluation of ATED probes for determination of WRN helicase activity. A) The design of ATED probes with different bubble sizes and duplex lengths. B) The fluorescence responses of ATED probes with different bubble sizes to WRN. C) The fluorescence responses of ATED probes with different duplex lengths to WRN. D) Normalized fluorescence response of ATED probes to WRN. The concentration of the probe is 50 nm, and WRN is 27 nm. The columns are expressed as mean ± SD (n = 3).

Based on this observation, we then designed the ATED probe for WRN. During the unwinding process, WRN is expected to initially recognize and bind to the single‐stranded bubble region before sequentially unwinding the duplex DNA on both sides of the bubble in the 3ʹ–5ʹ direction.^[^ ^5a^ ^]^ In the ATED probe (**Figure** **3**A), the central bubble region could act as an anchor, and the double helix region with a fluorescence label might serve as a reporter for the unwinding activity. As the flexibility of the central bubble and double helix sequences might significantly influence the WRN unwinding reaction and consequently affect the probe's sensitivity, diverse probes with differing bubble area sizes (from 6 to 10 nt) and double helix area lengths (from 11 to 23 bp) were designed to achieve better detection sensitivity to WRN (Figure 3A; Table S1, Supporting Information). Obviously, both bubble sizes and double helix lengths significantly influence the sensitivity of the ATED probe for WRN. The fluorescence response of probe with an 8 nt bubble (**B8D19)** was much stronger than that of the 6 nt bubble **(B6D19)**, and slightly stronger than that of the 10 nt bubble **(B10D19)**. These results suggest that WRN prefers unwinding probes with a bubble size of 8 nt, which may possess an optimal structure for recruiting WRN (Figure 3B). This might refer to the different ability of bubble to recruit WRN. Notably, the sensitivity of ATED probes with different lengths of double helix to WRN was surprising. In a previous study, we found WRN and BLM unwinding forked DNA faster when the double helix region is shorter.^[^ ^13^ ^]^ However, in this case, when WRN unwound bubble DNA with different double helix regions, probe **B8D19** with 19 bp duplex region exhibited more sensitivity to WRN than probe **B8D11** with 11 bp duplex region. Additionally, probes with 15 and 23 bp duplex regions (**B8D15** and **B8D23)** exhibited similar sensitivity, but the responses of these probes were much weaker than **B8D11** (Figure 3C,D). These findings suggest that WRN exhibits a more complex unwinding mechanism when interacting with bubble DNA compared to forked DNA.

### 2.2 MD Simulation‐Assisted Optimization of ATED Probes for WRN

**Figure 4:**
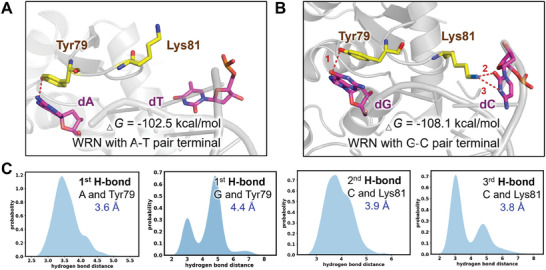
The interaction between WRN and different DNA helices were evaluated by 50 ns MD simulations. The structure was conducted from crystall structure (PDB ID: 3AAF). A) the hydrogen bonds formed between WRN and DNA with GC pairs. B–D) the distances of the hydrogen bonds between WRN and different base pairs.

To further optimize the ATED probe sequence, we employed molecular dynamics (MD) simulations to investigate the interactions between WRN and DNA duplexes with different base pair compositions. In the crystal structure of the WRN‐DNA complex, the protein bound the helix DNA, while the residue Tyr79 and Lys81 induced the unwinding of the terminal base pair. To predict the preference of WRN for DNA base pair, different base pair models were conducted from the crystal structure. One with an A‐T base pair on the DNA terminal (A‐T‐model), and the other with a G‐C base pair on the DNA terminal (G‐C‐model). After MD simulation to the equilibrium state, we found that in the A‐T model, the terminal A‐T base only formed one hydrogen bond with Tyr79 of WRN (**Figure** **4**A). In the G‐C model, three hydrogen bonds were observed. One was formed between Tyr79 and G base, the other two were formed between Lys81 and C base (Figure 4B). The above observations indicated the favorable free energy contribution arose from the C base. The distances of the atom pairs that may form hydrogen bonds are monitored. As evident from Figure 4C, the newly formed hydrogen bonds remained stable over 50 ns of MD simulations.

**Figure 5:**
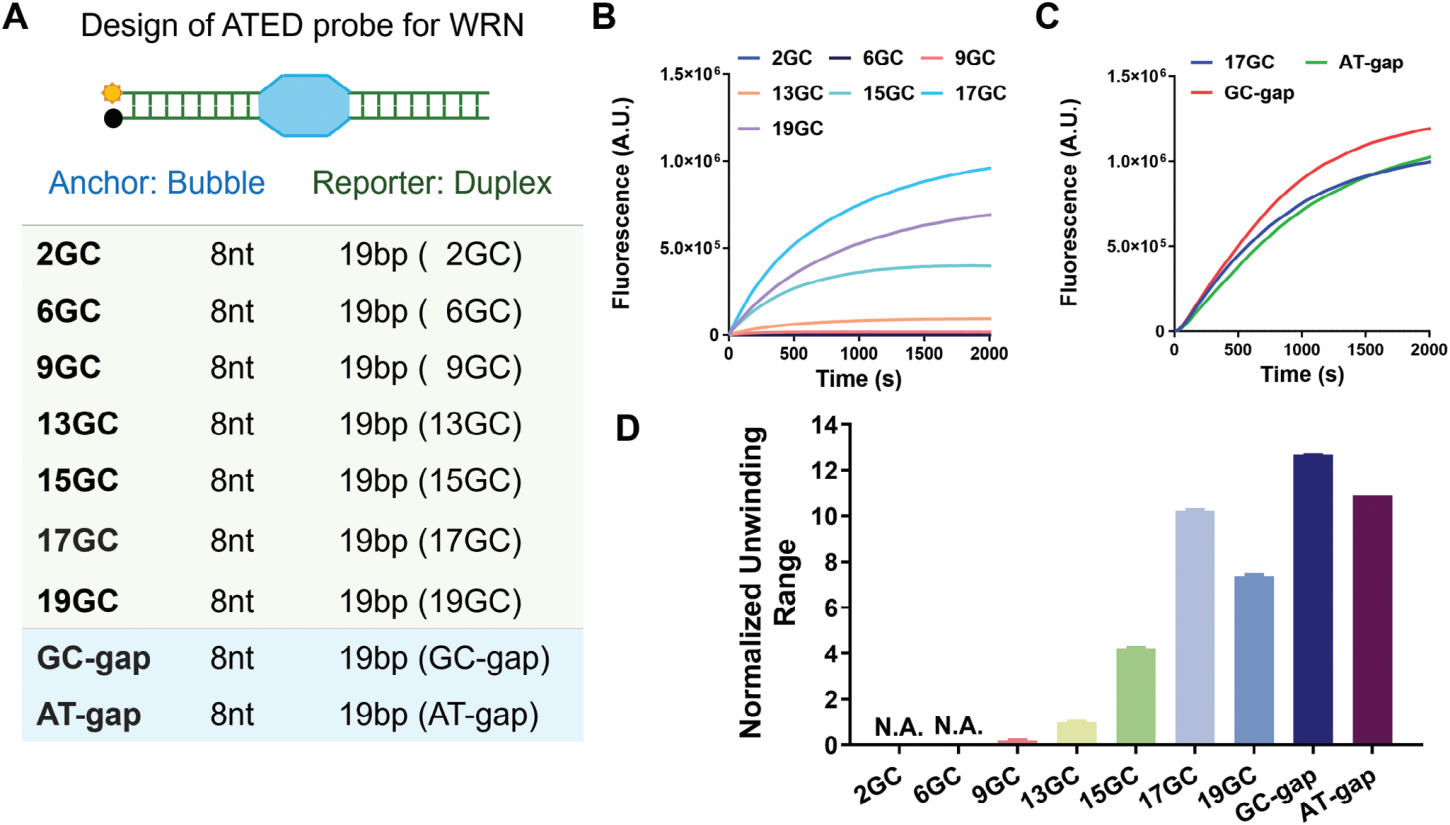
Optimization of ATED probes for determination of WRN helicase activity. A) The design of ATED probes with different duplex sequence. B) The fluorescence responses of ATED probes with different GC ratio to WRN. C) The fluorescence responses of ATED probes with different interface base between bubble and duplex to WRN. D) Normalized fluorescence response of ATED probes to WRN. The concentration of probe is 50 nm, and WRN is 27 nm. The columns are expressed as mean ± SD (n = 3). N.A. represents the signal was not above the threshold of background noise.

To quantify the energetic differences, MM/PBSA free energy calculations were performed for both models. The total MM/PBSA values were averaged over the 1–50 ns timeframe. The average binding free energy for the A‐T‐model was ‐102.5 kcal mol^−1^, while that for the G‐C‐model was −108.1 kcal mol^−1^, indicating a clear energetic advantage for the GC mutation. Additionally, the free energy contribution of a single base toward protein binding was calculated. It is obvious that the C base was more favorable in the unwinding pocket than the T base, with the average free energy contribution of the T base being ‐1.52 kcal mol^−1^, and the C base contributing −4.53 kcal mol^−1^. Based on the MD simulations, the G‐C base pair is more favorable for interacting with the unwinding pocket of WRN protein than the A‐T base pair. Based on these findings, we optimized the ATED probes by increasing the GC content in the duplex region (**Figure** **5**A). As shown in Figure 5B, GC‐rich ATED probes **19GC**, **17GC,** and **15GC** exhibited a much stronger response to WRN than other probes with lower GC content (**2GC**, **6GC**, **9GC,** and **13GC**). Except for **19GC**, the increase of GC ratio significantly increased the fluorescence response of the ATED probe to WRN. The GC‐rich probe **17GC** with 17 bp GC among 19 bp duplex region exhibited a response signal 10.2 times stronger than **B8D19 (13GC)**, indicating the success in the probe optimization. In addition, we also evaluate the influence of adjacent bases on WRN unwinding efficiency. Interestingly, the probe with a GC base pair adjacent to the bubble region showed slightly higher response to WRN, as compared to the probe with at least one AT base pair near the bubble (Figure 5C,D). Above all, the sequence optimization of the ATED probe significantly improved their detection performance to WRN unwinding activity. The optimized probe **17GC** with 8 nt bubble, 19 bp GC‐rich duplex DNA was selective for further evaluation. Moreover, our findings indicated that WRN exhibits a strong preference for GC sequences. This observed phenomenon contrasts with previous reports,^[^ ^22^ ^]^ warranting further investigation into the underlying reason.

### 2.3 Sensitive and Specific Detection of WRN by ATED Probe 17GC In Vitro

**Figure 6:**
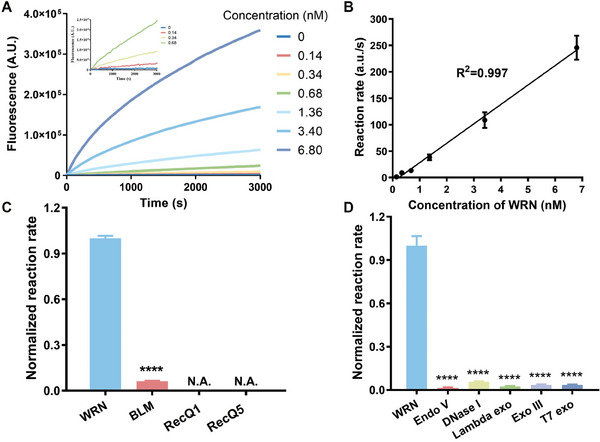
(A) The fluorescence responses of the probe (50 nm) to WRN from 0.14 to 6.8 nm. Inset: The fluorescence intensity over time to low concentrations of the WRN from 0.14 to 0.68 nm. B) The linear relationship between the rate of fluorescence increases and the WRN concentration from 0.14 to 6.8 nm. (WRN in the range of 0–0.7 nm is shown in the inset). C) The specificity of the probe toward WRN over other helicases (25 nm). D) The fluorescence responses of the probe toward WRN (4.0 U L^−1^) over other nucleases (DNase I: 1000 U L^−1^; Endo V: 2000 U L^−1^; Lambda exo: 10 000 U L^−1^; Exo III: 750 U L^−1^; T7 exo: 2000 U L^−1^. Biological replicates (n = 3) were taken. The data are presented as mean ± SD, and statistical significance is determined by the *t* test as (ns) not significant, (*) *P* < 0.05, (**) *P* < 0.01, and (***) *P* < 0.001, comparing to the first group. N.A. represents the signal was not above the threshold of background noise.

The fluorescence response ability of the ATED probe was studied by adding various concentrations of WRN into the reaction system. As depicted in **Figure** **6**A,B, the fluorescence intensity enhanced gradually with increasing WRN concentration. A strong linear relationship was observed between the rate of fluorescence increase and WRN concentration in the range of 0.14–6.8 nm. The limit of detection (LOD) was determined to be as low as 33.5 pm. To the best of our knowledge, this LOD surpasses that of the existing approaches (Table S4, Supporting Information). The remarkable sensitivity of this method can be attributed to the structural designs of the ATED probe.

The selectivity of the WRN assays was assessed by examining the anti‐interference capability of the bubble probe against three homologous helicases from RecQ family (RecQ1, BLM, and RecQ5). A robust fluorescence response was exclusively observed with WRN, while no detectable unwinding activity was observed for the other helicases (Figure 6C). In addition, DNA molecular probes are susceptible to degradation in real biological environments because of the nonspecific action of other potentially coexisting nucleases in cells, including DNase I, Endonuclease V, Lambda exonuclease, Exonuclease III, and T7 exonuclease. Consequently, further testing was conducted to determine whether the interference of these nucleases could be resisted by the probe. As illustrated in Figure 6D, there was no significant fluorescence increase for these enzymes. These results indicate the probe's high selectivity for WRN and its stability in biological environments.

### 2.4 Visualization of WRN Unwinding Activity by ATED Probe 17GC in Cancer Cells

**Figure 7:**
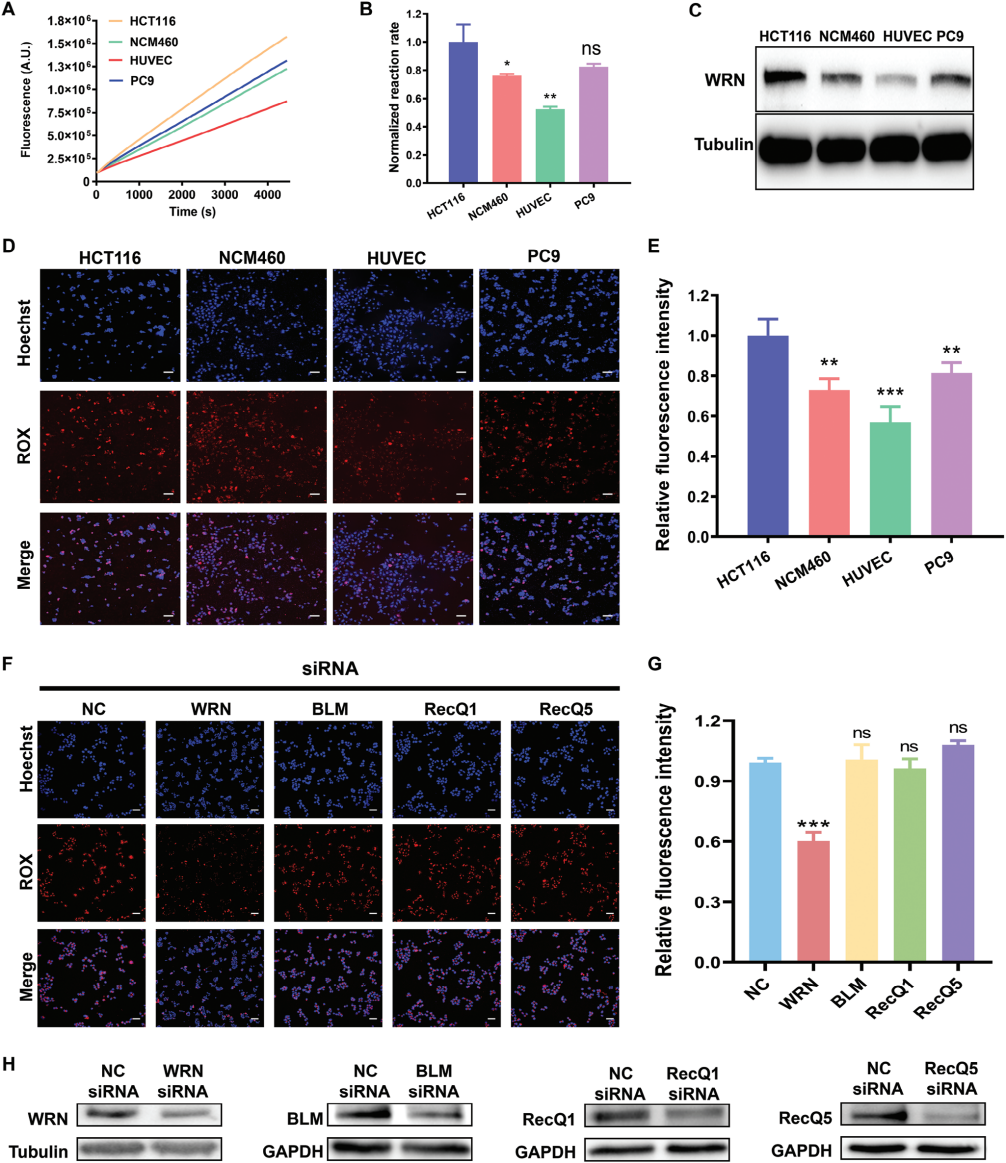
A) Fluorescence response and B) reaction rate of ATED probe to WRN in cell lysates from various cells. C) Western blot analysis of the WRN expression level in various cell lysates. D) Fluorescence imaging of ATED probe in different living cells. E) The corrected fluorescence intensity of the probe in various cells. F) Imaging and G) relative fluorescence intensity of ATED probe in HCT116 cells treated with siRNA Negative control (NC), WRN, BLM, RecQ1 and RecQ5. H) Western blot analysis of knockdown efficiency of siRNA treatment in HCT116 cells. The probe concentration is 10 nm, the scale bar is 100 µm. Biological replicates (n = 3) were taken. The data are presented as mean ± SD, and statistical significance is determined by the *t* test as (ns) not significant, (*) *P* < 0.05, (**) *P* < 0.01, and (***) *P* < 0.001, comparing to the first group.

Given that WRN is a critical anticancer target, the development of a technique to evaluate WRN inhibitors holds significant importance in cancer therapy. Currently, the effects of inhibitors on helicase activity are primarily assessed using biochemical screening assays.^[^ ^11^, ^23^ ^]^ However, these assays often fail to accurately reflect helicase activity in living cells. Thus, a reliable method for characterizing WRN activity in cellular contexts is urgently needed.^[^ ^24^ ^]^ To evaluate WRN activity in cells, the ATED probe **17GC** was delivered into HCT116 cells using Lipofectamine 3000 transfection reagent. The concentration and incubation time of the probe in living cells were optimized (Figures S1 and S2, Supporting Information). In addition, the transfection was confirmed to cause no cytotoxicity to cells (Figure S3, Supporting Information). The ability of the ATED probe to detect WRN activity was assessed first in various cell lysates, such as HCT116, NCM460, PC9, and HUVEC (**Figure** **7**A,B). The western blot analysis confirmed WRN expression levels in the lysates, which were consistent with the fluorescence signals of probes (Figure 7C). Then the ATED probes were transfected into living cells for intracellular imaging (Figure 7D). To eliminate the impact of transfection efficiency in different cells, the fluorescence signal was corrected using a probe without a quenching molecule (C‐Probe) (Figure S4, Supporting Information). The corrected fluorescence intensity of WRN activity in each cell type was then computed and shown in Figure 7E.

To further show the unwinding roles of WRN in the operation of the probe in living cells, a target siRNA of WRN was used to knockdown WRN expression in HCT116 cells (WRN siRNA group), whereas a non‐target siRNA served as the negative control (NC siRNA group). Confocal fluorescence images indicated a 40% decrease in fluorescence intensity in cells pre‐treated with target siRNAs compared with the NC siRNA group (Figure 7F,G). Furthermore, the western blot analysis findings indicated that ≈50% of WRN in HCT116 cells was knocked out (Figure 7H). The data also aligned with the findings obtained from the cell lysate assay and previous studies.^[^ ^25^ ^]^ In addition, we then evaluate the responses of **17GC** to other RecQ helicases, including BLM, RecQ1, and RecQ5 by siRNA knockdown treatments (Figure 7H). Consistent with in vitro performance, the cellular signal of **17GC** did not change upon other RecQ helicases (Figure 7F,G). These findings indicate that the probe accurately reveals WRN activity in living cells with high selectivity.

**Figure 8:**
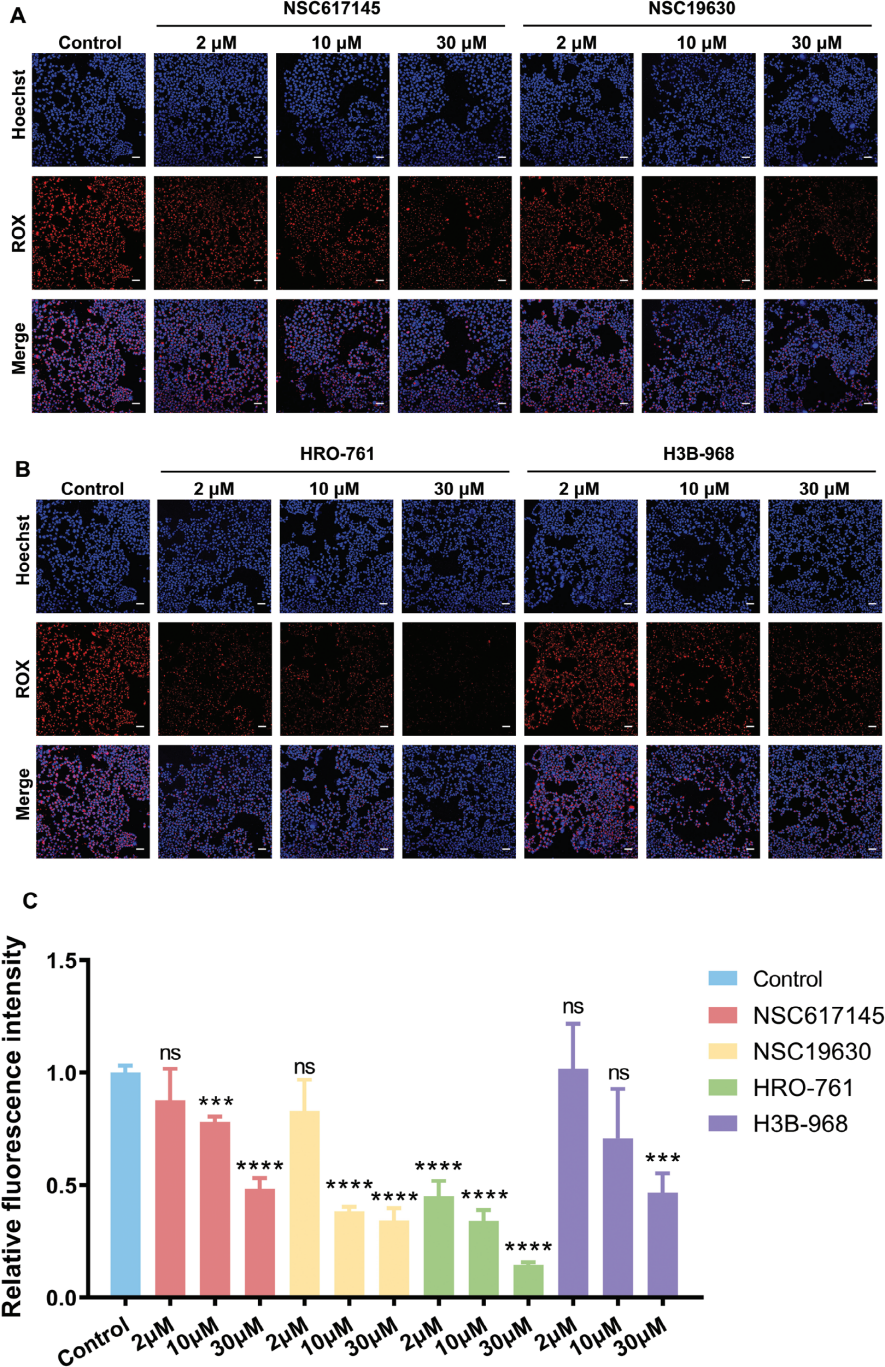
A) Imaging of ATED probe in HCT116 cells treated with NSC167145 and NSC19630. B) Imaging of ATED probe in HCT116 cells treated with HRO‐761 and H3B‐968. C) The relative fluorescence intensity of ATED probe in HCT116 cells treated with inhibitors. The probe concentration is 10 nm, the scale bar is 100 µm. Biological replicates (n = 3) were taken. The data are presented as mean ± SD, and statistical significance is determined by the *t* test as (ns) not significant, (*) *P* < 0.05, (**) *P* < 0.01, and (***) *P* < 0.001, comparing to the first group.

It is reported that inhibiting WRN is a potential strategy for microsatellite instability (MSI) cancer therapy.^[^ ^6a,c^ ^]^ Precise evaluation of WRN inhibitory efficiency in living cells might be promising for WRN inhibitor development. Thus, we assessed the potential of ATED probe **17GC** for evaluating WRN inhibitors. The inhibitory effects of the small‐molecule WRN inhibitors, including **NSC617145**, **NSC19630**, **HRO‐761**, and **H3B‐968,** on WRN activity in HCT116 cells were investigated by ATED probe. As illustrated in **Figure** **8**, the intracellular fluorescence signal significantly decreased following inhibitor treatment. This reduction in fluorescence was concentration‐dependent, reflecting a progressive decline in WRN enzymatic activity induced by the inhibitors. Among the tested compounds, **HRO‐761** demonstrated the strongest inhibitory effect, while **NSC617145**, **NSC19630**, and **H3B‐968** exhibited weaker effects. Notably, these cellular findings were consistent with the in vitro results, indicating that the ATED probe exhibits specificity and accuracy in screening and evaluating small molecule inhibitors for WRN. Furthermore, to explore the intracellular localization of ATED probes, we employed confocal microscopy to obtain higher‐resolution images. The results (Figure S5, Supporting Information) revealed efficient nuclear transfection of the probe, as evidenced by a clear nuclear localization signal. As WRN is primarily a nuclear protein, the co‐localization of ATED probe with WRN supports its nuclear localization. The cell imaging method by ATED probe **17GC** for WRN activity determination might be developed into an HTS WRN inhibitor screening assay in the future.

## 3 Discussion

DNA helicases play pivotal roles in maintaining genomic integrity by unwinding DNA and resolving a variety of structured nucleic acids. This critical function has made them attractive drug targets, especially in cancer therapy. While numerous methods have been developed to detect helicase activity in vitro and exhibited good performance in the detection of both ATPase and unwinding activity of helicase,^[^ ^12^, ^13^ ^]^ monitoring the unwinding activity of a specific helicase within living cells remains a significant challenge. This limitation arises from the duplex DNA substrates can be recognized and processed by multiple helicases in the cellular environment, making it difficult to achieve specificity. Some recent studies found duplex probes could sense the DNA metabolism process in cells.^[^ ^26^ ^]^ In this study, we proposed a design strategy for the ATED probe, which enhances the specificity of DNA substrate by introducing an “anchor” unit to recruit a target helicase and assess helicase unwinding activity both in vitro and in cellular contexts.

We focus on the promising anticancer target WRN. Following sequence optimization, we successfully achieved highly sensitive detection of intracellular WRN activity using the ATED DNA fluorescent probe **17GC** for the first time. During the in vitro optimization process, we found that WRN exhibits a preference for unwinding GC‐rich sequences, and we employed molecular simulation methods to elucidate the underlying mechanism. In the cellular imaging experiments, the ATED probe demonstrated a sensitive and specific response to WRN activity across different cell types, effectively distinguishing between WRN activity and various cell lines, with the potential for detecting low‐abundance WRN. Furthermore, this ATED probe facilitates the assessment of small molecule inhibitors’ activity at the cellular level, providing a valuable approach for high‐throughput screening and evaluation of small molecule compounds in cells. However, it should be noted that although treatments of WRN siRNA or inhibitors significantly reduced most fluorescence of ATED probe **17GC** in cells while treatment of other RecQ helicase siRNA did not, it is unavoidable that the interaction between WRN and ATED probes will be complicated in cells. The recruitment of WRN to the ATED probe **17GC** still will possibly be affected by other bubble‐binding proteins via competitive binding, cooperative binding, or other unexpected effects. In addition, it desired to further evaluate the design principle of “anchor” and “reporter” in the ATED probe, and also extend its application to other helicases. For example, the “ROX” label might be able to be replaced by the fluorescent binder.^[^ ^27^ ^]^ For helicases with low abundance, the nano‐based signal amplification method might also cooperate.^[^ ^28^ ^]^

Overall, this study establishes the ATED probe 17GC as a robust platform for detecting WRN activity in vitro and in living cells for the first time. By addressing the challenges of specificity and intracellular imaging, the probe provides a foundation for advancing the understanding of WRN biology and for facilitating the development of WRN‐targeted therapies. Furthermore, the design principles applied to the ATED probe could be extended to the development of probes for other DNA helicases, thereby broadening the scope of this methodology for helicase research and drug discovery. Future studies could focus on adapting the ATED probe for high‐throughput screening (HTS) applications, which would accelerate the identification of novel WRN inhibitors for clinical use.

## 4 Experimental Section

### Materials and Reagents

The DNA oligonucleotides and siRNAs used in this study (Tables S1 and S3, Supporting Information) were synthesized and purified (HPLC grade) by Generay Biotechnology Co., Ltd. (China). Recombinant WRN and Bloom syndrome helicase (BLM) proteins with complete helicase activity were expressed in Sf9 system and obtained from Sino Biological (China). Recombinant RecQ1 and RecQ5 proteins with complete helicase activity were expressed in BL21 and purified by a Ni column (GE HealthCare Technologies Inc. USA). The following nucleases and their buffers were obtained from New England Biolabs Inc. (USA): deoxyribonuclease I (DNase I), endonuclease V (Endo V), exonuclease III (Exo III), lambda exonuclease (Lambda exo), and T7 exonuclease (T7 exo). The WRN inhibitors NSC19630, NSC617145, HRO‐761 and H3B‐968 were purchased from Med Chem Express Inc. (USA). ATP was obtained from Sigma Chemical Co. (USA). Lipofectamine 3000 transfection reagent, Opti‐MEM, Dulbecco's modified Eagle's medium (DMEM), RPMI 1640 medium, fetal bovine serum, 0.25% trypsin, and penicillin–streptomycin were obtained from Thermo Fisher Scientific Inc. (USA). Hoechst 33 342 was obtained from Beyotime Biotechnology Co., Ltd. (China).

### Filter‐Binding Assay

Filter‐binding assays were performed as follows. In brief, a nylon membrane was placed beneath a nitrocellulose membrane to trap any DNA not retained on the nitrocellulose. The nitrocellulose membrane was then treated with 0.5 m KOH for 10 min at 4 °C and washed with 0.5 × TB prior to use. Biotinylated DNA (20 nm) was incubated with peptides or proteins at 37 °C for 3 h in binding buffer (10 mm Tris‐HCl, 60 mm NaCl, 2 mm MgCl_2_, pH 7.4). All samples were applied to the membrane under vacuum and washed with binding buffer. The cross‐linking reaction was carried out under UV irradiation at 265 nm for 90 s. Biotinylated DNA was detected using a Chemiluminescent Nucleic Acid Detection Module Kit (Thermo Fisher Scientific, USA). The grey levels of the dots were quantified using Quantity One. The *K*_D_ value was evaluated by fitting the data to a Hill model. The obtained data were analyzed using GraphPad Prism (GraphPad Software, San Diego, CA).

### Determination of WRN Activity by ATED Probes In Vitro

In a 200 µL PCR tube, 5.0 µL of the ATED probe (0.5 µm, Table S1, Supporting Information), 5.0 µL each of 10x reaction buffer (Table S2, Supporting Information) and 5.0 µL of 10 mm ATP were mixed with 30 µL of ddH_2_O. The reaction was initiated by adding 5.0 µL of WRN protein to the mixture. The fluorescence intensity of the reaction solution was monitored using an ABI 7500 Real‐Time Fluorescent PCR system (Thermo Fisher Scientific, USA). The thermal program consisted of 31 seconds per cycle at 25 °C, with fluorescence measurements conducted at the end of each cycle. The excitation and emission were set at 588 and 608 nm, respectively. The fluorescence intensity curve over time was used to calculate the fluorescence increase rate, providing a quantitative measure of WRN unwinding activity. The fluorescence increasing rate of the control group was normalized to 1.0. The formula for signal normalization was R = X/Ȳ. “R” is the normalized value of X relative to Ȳ, where “X” is the sample value and “Ȳ” is the average of the values used for comparison.

### Molecular Dynamics Simulation

The WRN‐DNA complex (PDB ID: 3AAF)^[^ ^29^ ^]^ was obtained from the Protein Data Bank and prepared using Maestro software. Since the crystal structure contained an AT terminal pair, they were also mutated to GC pairs to study the different interactions with the protein. Both the unmutated and mutated structures were prepared for subsequent MM/PBSA calculations. Each complex was solvated in a TIP3P water box, neutralized with appropriate Na^+^ or Cl^−^ counterions. The Amber ff14SB force field was used to parameterize the protein, while the required force field parameters for DNA were loaded. Molecular dynamics (MD) simulations were performed using AMBER20. Initially, a four‐step minimization was carried out, with each step employing 2500 steps of steepest descent and 2500 steps of conjugate gradient minimization, gradually releasing the positional restraints. The systems were then heated to 300 K over 50 ps using Langevin dynamics. Density equilibration with weak restraints on the complex was followed by 100 ps constant pressure equilibration, maintaining 1 atm pressure using the Parrinello‐Rahman barostat. During these preliminary NVT and NPT equilibrations, a restraint weight of 10 kcal mol^−1^ Å^−2^ was applied. Subsequently, 50 ns production MD simulations were performed for each system under periodic boundary conditions, at 1 atm pressure and 300 K temperature, using the NPT ensemble. The SHAKE algorithm constrained bonds involving hydrogen atoms, allowing a 2 fs time step. Long‐range electrostatic interactions were treated with the particle mesh Ewald (PME) method, using an 8 Å cutoff.

### Cell Culture and siRNA Treatment

The human colon cancer cell line HCT116 and human normal colon epithelial cell line NCM460 were cultured in RPMI 1640 medium supplemented with 10% (v/v) fetal bovine serum (FBS) and 1% (v/v) penicillin–streptomycin (P/S). The human non‐small‐cell lung cancer cell line PC9 and the human umbilical vein endothelial cell line HUVEC were cultured in DMEM with 10% (v/v) FBS and 1% (v/v) P/S. In addition, all cells were cultured at 37 °C in an HF100 carbon dioxide incubator (Heal Force, China) under a humidified atmosphere of 5% CO_2_. For siRNA treatment, lipofectamine 3000 transfection reagent was used to transfect siRNA into cells following the manufacturer's protocol. After 24 h of transfection, the cells were then applied for cell imaging or western blotting assay.

### Western Blot (WB) Assay

HCT116 cells were seeded in a 6‐well plate (Nest, China) and cultured for 40 h. Lipofectamine 3000 transfection reagent (7.5 µL) was then used to transfect WRN siRNA (2.5 µg siRNA1 + 2.5 µg siRNA2) or NC siRNA (5 µg) into the cells. The same procedure was applied for knocking down other proteins (e.g., BLM, RecQ1, RecQ5) using their respective siRNAs. After 24 h of transfection, the cells were washed three times with ice‐cold PBS and then lysed on ice using RIPA Lysis Buffer with a protease inhibitor cocktail. The lysates were centrifuged at 12 000 rpm for 20 minutes at 4 °C, and the supernatants were collected for protein quantification using a BCA protein detection kit. Subsequently, equal amounts of proteins (25 µg) were loaded onto a gel for electrophoresis and transferred them to a polyvinylidene difluoride membrane (GE Healthcare, USA). The membrane was blocked with 5% fat‐free milk at room temperature for 2 h and then incubated overnight at 4 °C with primary antibodies against WRN (ABclonal, China), BLM (Signalway Antibody LLC, USA), RecQ1 (Proteintech, USA) or RecQ5 (Proteintech, USA). The membrane was then washed with 1x TBST three times and incubated with a secondary antibody Tubulin (ABclonal, China) or GAPDH (Proteintech, USA) at room temperature. A chemiluminescence (ECL) kit (Beyotime Biotechnology, China) and a Tanon 5200 chemiluminescence instrument (Tanon, China) were used to obtain the signal.

### High‐Content Imaging Analysis of WRN Activity in Live Cells

HCT116 cells were plated at 1.2 × 10^4^/well in a 96‐well plate with a glass bottom (Cellvis, USA, P96‐1.5H‐N) for 40 h. Other cell lines (NCM460, PC9, and HUVEC) were seeded at a suitable density in a 96‐well plate and cultured under the same conditions. For siRNA treatment, lipofectamine 3000 transfection reagent was used to transfect siRNA into the cells following the manufacturer's protocol. Furthermore, for assaying the inhibitory effects of the small‐molecule inhibitor for WRN, **NSC617145**, **NSC19630**, **HRO‐761**, and **H3B‐968** were respectively dissolved in the above 100 µL fresh medium containing 1.2 µL DMSO. Then, the culture medium in each well was removed, and the aforementioned mixture was added. After 2 h of incubation, the cells were washed with PBS, and 50 µL Hoechst 33 342 was introduced. Subsequently, an MetaXpress 1.0 high‐content cell imaging system was employed to image the cells. The fluorescence of Hoechst 33 342 and ROX in cells was imaged in their respective channels.

## Conflict of Interest

The authors declare no conflict of interest.

## Supporting information



Supporting Information

## Data Availability

The data that support the findings of this study are available in the supplementary material of this article.
