# Supplementary material for: Specific Monitoring the DNA Helicase Function via Anchor‐Embedded DNA Probe
Source: Adv Sci (Weinh). 2024 Dec 31;12(8):2413368. doi: 10.1002/advs.202413368 (PMC11848566; doi:10.1002/advs.202413368)
Supplement: Supplementary file 1 — Supporting Information [file ADVS-12-2413368-s001.docx]

Supporting Information for

Specific Monitoring the DNA Helicase Function via Anchor-Embedded DNA Probe

Keni Ning^1,#^, Xiaoyan Tang^1,#^, Zhe Li^2^, Liting Zhong^2^, Yingchen Zhou^2^, Jiaen Wang^2^, Wanyi Huang^2^, Han Zhang^1^, Jiajun Ke^1^, Tiangang Luan^3,4,*^, Shuo-Bin Chen^2,*^ Junqiu Zhai^1,*^

^1^School of Pharmaceutical Sciences, Guangzhou University of Chinese Medicine, Guangzhou 510006, China

^2^School of Pharmaceutical Sciences, Guangdong Provincial Key Laboratory of New Drug Design and Evaluation, Sun Yat-sen University, Guangzhou 510006, China

^3^School of Life Sciences, Sun Yat-sen University, Guangzhou, 510275, China

^4^School of Environmental and Chemical Engineering, Wuyi University, Jiangmen, 529020, China

#These authors contributed equally.

*Corresponding authors: E-mail: cesltg@mail.sysu.edu.cn (T. Luan), chenshb8@mail.sysu.edu.cn (S. Chen), jqzhai@gzucm.edu.cn (J. Zhai)

**Contents**

1. Enzymatic reactions in vitro 3
2. Assay of WRN activity in cell lysates 3
3. Cell culture 3
4. SiRNA knockdown 3
5. Protein extraction and western blot (WB) of different cells 4
6. Table S1. Sequences of the oligonucleotides used in this work^a^. 5
7. Table S2. The composition and pH of the reaction buffers for the enzymes studied in this work. 6
8. Table S3. Sequences of the siRNAs used in this work^b^. 6
9. Table S4. Linear range and LOD of WRN in the different methods. 7
10. Figure S1. Representative fluorescence images of HCT116 cells after incubation with various concentrations of ATED probe for 2 h. (The scale bar is 100 μm) 7
11. Figure S2. Representative fluorescence images of HCT116 cells after incubation with 10 nM ATED probe for different time. (The scale bar is 100 μm) 8
12. Figure S3. (A) Cytotoxicity evaluation and (B) Cell density measurement of the ATED probe across a concentration range from 0 to 200 nM. 8
13. Figure S4. Transfection efficiency diagram of C-probe (10 nM) in various cells. (The scale bar is 100 μm) 9
14. Figure S5. Imaging of ATED probe (10 nM) in HCT116 cells. The scale bar is 10 μm. 9

# Enzymatic reactions in vitro

**Annealing of DNA probes:** DNA probes was prepared by annealing two complementary oligonucleotides (10 μM each, see the sequences in Table S1, Supporting Information) in a buffer containing 10 mM Tris-HCl (pH 7.4) and 120 mM NaCl. Annealing program: all samples were heated at 95°C for 5 min, gradually cooled to room temperature.

**Helicases:** In a 200 μL PCR tube, 5.0 μL each of 10× reaction buffer (Table S2, Supporting Information), 10 mM ATP and 0.5 μM probe were thoroughly mixed with 30 μL of ddH_2_O. Then 5.0 μL of helicase was introduced into the mixture to initiate the reaction.

**Nucleases:** In a 200 μL PCR tube, 5.0 μL each of 10× reaction buffer (Table S2, Supporting Information) and 0.5 μM probe were thoroughly mixed with 35 μL of ddH_2_O. Then 5.0 μL of nuclease was introduced into the mixture to initiate the reaction.

The detection process followed the procedure described as before.

# Assay of WRN activity in cell lysates

In a 200 μL PCR tube, 5.0 μL of 10× reaction buffer (Table S2, Supporting Information), 5.0 μL of 10 mM ATP, and 5.0 μL of 2 μM probe were thoroughly mixed with 25 μL of ddH_2_O. Subsequently, 10.0 μL of cell lysate (0.8 mg/mL) was introduced into the mixture to initiate the reaction. The total volume of the reaction system was 50 μL. The detection process followed the same procedure as described before.

# Cell culture

We cultivated the human colon cancer cell line HCT116 and human normal colon epithelial cell line NCM460 in RPMI 1640 medium supplemented with 10% (v/v) fetal bovine serum (FBS) and 1% (v/v) penicillin–streptomycin (P/S). We separately cultivated the human non-small-cell lung cancer cell line PC9 and the human umbilical vein endothelial cell line HUVEC in DMEM with 10% (v/v) FBS and 1% (v/v) P/S. In addition, all cells were cultured at 37°C in an HF100 carbon dioxide incubator (Heal Force, China) under a humidified atmosphere of 5% CO_2_.

# Protein extraction and western blot (WB) of different cells

The freeze-thaw approach was employed to extract the total protein in the cells. Collected cells were suspended in PBS containing a protease inhibitor cocktail and then frozen at −80°C for 30 min. Subsequently, the cells were slowly thawed at room temperature. The aforementioned steps were repeated three times. The cells were then centrifuged at 12 000 rpm for 20 min at 4°C, and the resulting supernatant was collected as cell lysate. A BCA protein detection kit was employed to determine the protein concentration. The WB experiments conducted on various cells yielded results consistent with those described above.

# Table S1. Sequences of the oligonucleotides used in this work^a^.

| **Sequences（5’-3’）** | | | | | | |
| --- | --- | --- | --- | --- | --- | --- |
| **Name** | **Chain** | | **Label** | **Duplex 1** | **Bubble** | **Duplex 2** |
| **B6D19** | A | 5-ROX | | GCGCGGAAGCTTGGCTGCA | GAATTG | CTAGCGGGAATTCGGCGCG |
|  | B | 3-BHQ2 | | CGCGCCGAATTCCCGCTAG | TGGCCT | TGCAGCCAAGCTTCCGCGC |
| **B8D19**  **（13GC）** | A | 5-ROX | | GCGCGGAAGCTTGGCTGCA | GAATATTG | CTAGCGGGAATTCGGCGCG |
|  | B | 3-BHQ2 | | CGCGCCGAATTCCCGCTAG | TGGCGCCT | TGCAGCCAAGCTTCCGCGC |
| **B10D19** | A | 5-ROX | | GCGCGGAAGCTTGGCTGCA | GGAATATTGG | CTAGCGGGAATTCGGCGCG |
|  | B | 3-BHQ2 | | CGCGCCGAATTCCCGCTAG | TTGGCGCCTT | TGCAGCCAAGCTTCCGCGC |
| **B8D23** | A | 5-ROX | | TACTGCGCGGAAGCTTGGCTGCA | GAATATTG | CTAGCGGGAATTCGGCGCGTCAT |
|  | B | 3-BHQ2 | | ATGACGCGCCGAATTCCCGCTAG | TGGCGCCT | TGCAGCCAAGCTTCCGCGCAGTA |
| **B8D15** | A | 5-ROX | | GGAAGCTTGGCTGCA | GAATATTG | CTAGCGGGAATTCGG |
|  | B | 3-BHQ2 | | CCGAATTCCCGCTAG | TGGCGCCT | TGCAGCCAAGCTTCC |
| **B8D11** | A | 5-ROX | | GCTTGGCTGCA | GAATATTG | CTAGCGGGAAT |
|  | B | 3-BHQ2 | | ATTCCCGCTAG | TGGCGCCT | TGCAGCCAAGC |
| **2GC** | A | 5-ROX | | AATTATAATATTTATTGCA | GAATATTG | CTAGTATTAATTAATTTAA |
|  | B | 3-BHQ2 | | TTAAATTAATTAATACTAG | TGGCGCCT | TGCAATAAATATTATAATT |
| **6GC** | A | 5-ROX | | GATCATAAGCTTTATTGCA | GAATATTG | CTAGTAGGAATTAATCTAG |
|  | B | 3-BHQ2 | | CTAGATTAATTCCTACTAG | TGGCGCCT | TGCAATAAAGCTTATGATC |
| **9GC** | A | 5-ROX | | GCGCATAAGCTTGATTGCA | GAATATTG | CTAGTAGGAATTCATCGCG |
|  | B | 3-BHQ2 | | CGCGATGAATTCCTACTAG | TGGCGCCT | TGCAATCAAGCTTATGCGC |
| **15GC** | A | 5-ROX | | GCGCGGAGCGCTGGCTGCA | GAATATTG | CTAGCGGGACGTCGGCGCG |
|  | B | 3-BHQ2 | | CGCGCCGACGTCCCGCTAG | TGGCGCCT | TGCAGCCAGCGCTCCGCGC |
| **17GC** | A | 5-ROX | | GCGCGGCGCGGCGGCTGCA | GAATATTG | CTAGCGCGGCGGGCGCGCG |
|  | B | 3-BHQ2 | | CGCGCGCCCGCCGCGCTAG | TGGCGCCT | TGCAGCCGCCGCGCCGCGC |
| **19GC** | A | 5-ROX | | GCGCGGCGCGGCGGCGGCC | GAATATTG | CCGGCGCGGCGGGCGCGCG |
|  | B | 3-BHQ2 | | CGCGCGCCCGCCGCGCCGG | TGGCGCCT | GGCCGCCGCCGCGCCGCGC |
| **GC-gap** | A | 5-ROX | | GCGCGGCGCGGCGGCTGAC | GAATATTG | CTAGCGCGGCGGGCGCGCG |
|  | B | 3-BHQ2 | | CGCGCGCCCGCCGCGCTAG | TGGCGCCT | GTCAGCCGCCGCGCCGCGC |
| **AT-gap** | A | 5-ROX | | GCGCGGCGCGGCGGCTGCA | GAATATTG | TCAGCGCGGCGGGCGCGCG |
|  | B | 3-BHQ2 | | CGCGCGCCCGCCGCGCTGA | TGGCGCCT | TGCAGCCGCCGCGCCGCGC |

^a^ROX is fluorescein and BHQ2 is Black Hole Quencher 2.

# Table S2. The composition and pH of the reaction buffers for the enzymes studied in this work.

| **Name** | **Buffer (1×)** | **pH (25℃)** |
| --- | --- | --- |
| WRN | 10 mM Tris-HCl, 60 mM NaCl, 1 mM MgCl_2_, 0.7% Triton X-100 | 7.4 |
| RECQ1 | 10 mM Tris-HCl, 60 mM NaCl, 1 mM MgCl_2_, 0.7% Triton X-100 | 7.4 |
| BLM | 10 mM Tris-HCl, 60 mM NaCl, 1 mM MgCl_2_, 0.7% Triton X-100 | 7.4 |
| RECQ5 | 10 mM Tris-HCl, 60 mM NaCl, 1 mM MgCl_2_, 0.7% Triton X-100 | 7.4 |
| Endonuclease V | 50 mM potassium acetate, 20 mM Tris-acetate, 10 mM magnesium acetate, 1 mM Dithiothreitol | 7.9 |
| Lambda  Exonuclease | 67 mM Glycine-KOH, 2.5 mM MgCl_2_, 50 µg/ml BSA | 9.4 |
| Exonuclease III | 10 mM Bis Tris Propane-HCl, 10 mM MgCl_2_, 1 mM Dithiothreitol | 7.0 |
| DNase I | 10 mM Tris-HCl, 2.5 mM MgCl_2_, 0.5 mM CaCl_2_ | 7.6 |
| T7 Exonuclease | 50 mM potassium acetate, 20 mM Tris-acetate, 10 mM magnesium acetate, 1 mM Dithiothreitol | 7.9 |

# Table S3. Sequences of the siRNAs used in this work^b^.

| **Name** | **Sequences（5’-3’）** |
| --- | --- |
| siRNA1 | AGUAAGAUGAAACCCUCCgt  tgUCAUUCUACUUUGGGAGG |
| siRNA2 | GCCUUAACAGUCUGGUUAAACtt  ttCGGAAUUGUCAGACCAAUUUG |
| NC siRNA | UUCUCCGAACGUGUCACGUtt  ttAAGAGGCUUGCACAGUGCA |

^b^The overhang bases are marked in red.

# Table S4. Linear range and LOD of WRN in the different methods.

| **Methods** | **Instrument** | **Linear range (nM)** | **LOD (nM)** |
| --- | --- | --- | --- |
| DNA Fluorescent Probe | qPCR | 0.14-6.8  (0.02-1.0 U/L) | 0.0335  (0.005 U/L) |
| Immunosorbent Assay | Well-plate reader | 0-2.5 | 0.12 |
| Chemiluminiscent EMSA | Gel imager | 0-2.5 | 0.16 |
| Fluorescent EMSA | Gel imager | 0-5000 | 1700 |
| Fluorescence Spectra | Spectrophotometer | 0-50 | 2.86 |

_
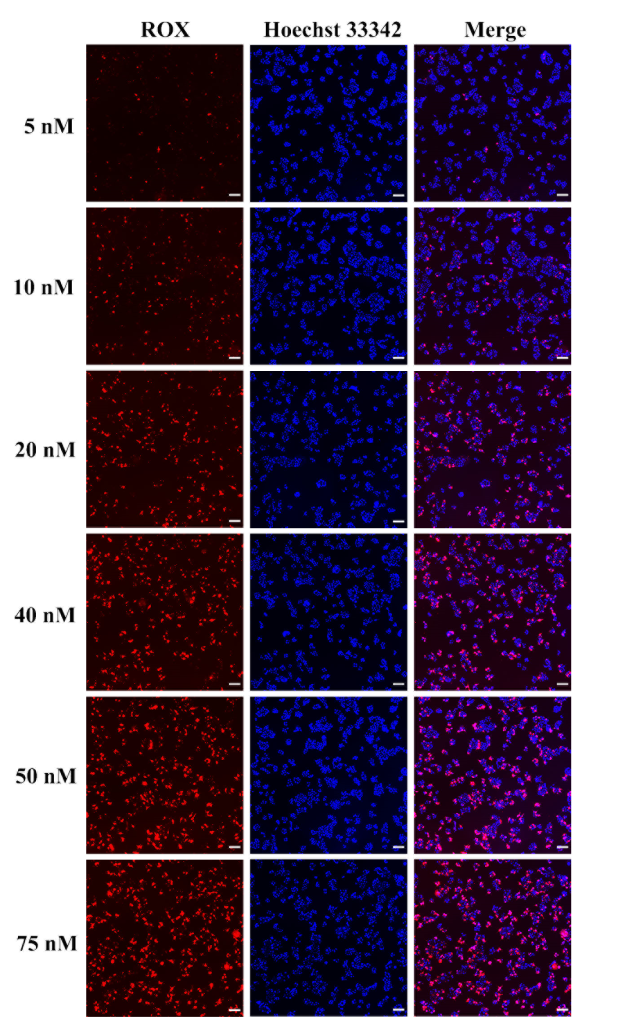
_

# Figure S1. Representative fluorescence images of HCT116 cells after incubation with various concentrations of ATED probe for 2 h. (The scale bar is 100 μm)


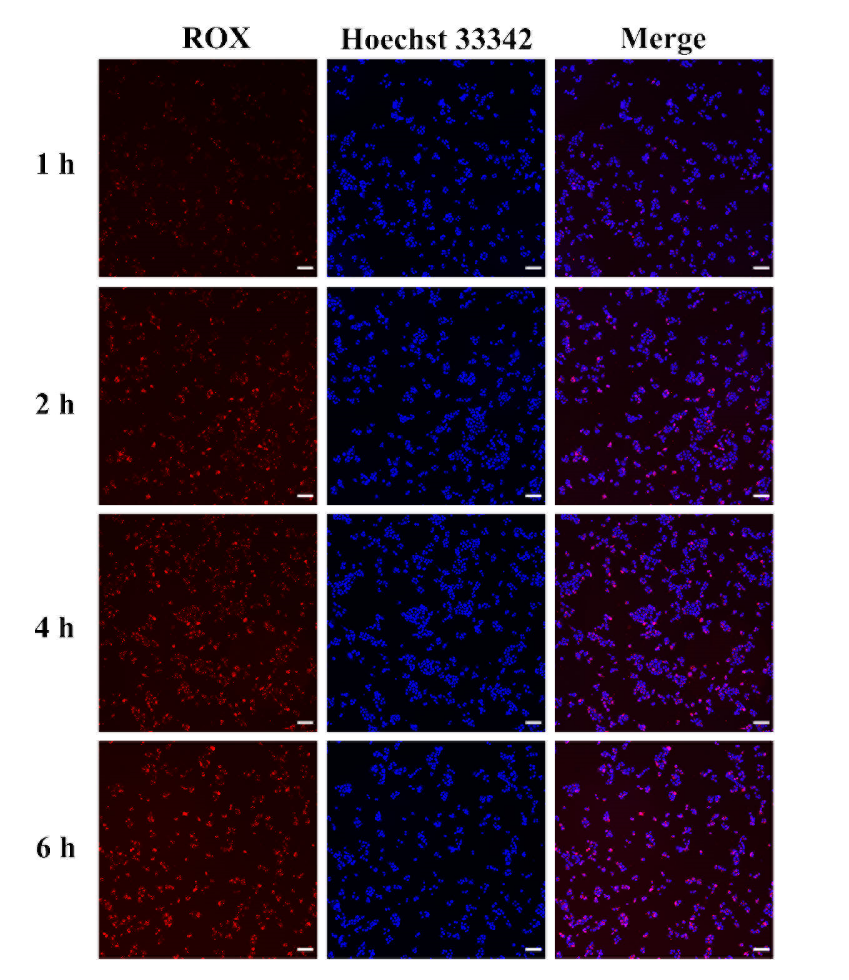


# Figure S2. Representative fluorescence images of HCT116 cells after incubation with 10 nM ATED probe for different time. (The scale bar is 100 μm)


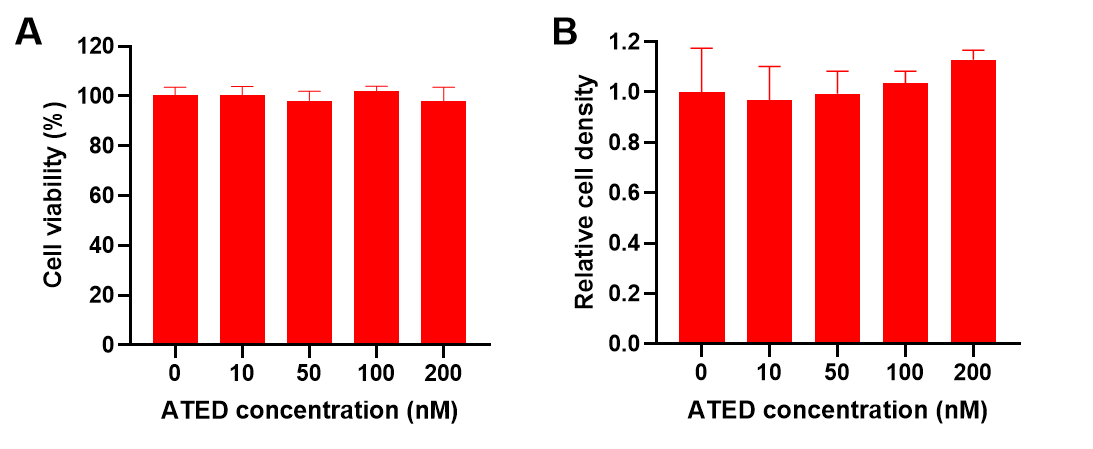


# Figure S3. (A) Cytotoxicity evaluation and (B) Cell density measurement of the ATED probe across a concentration range from 0 to 200 nM.


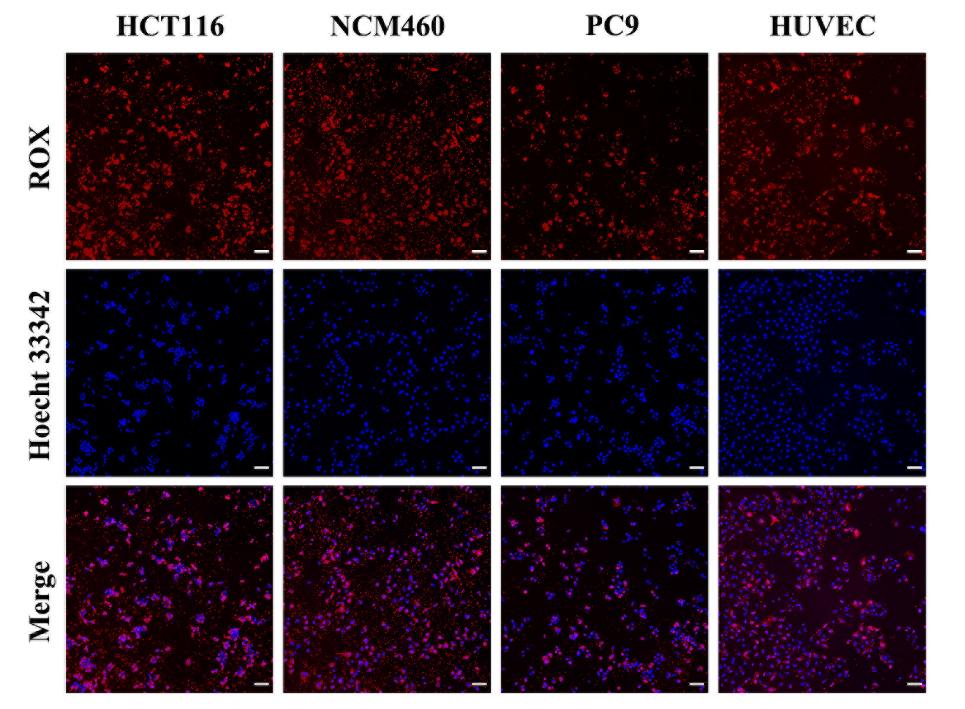


# Figure S4. Transfection efficiency diagram of C-probe (10 nM) in various cells. (The scale bar is 100 μm)


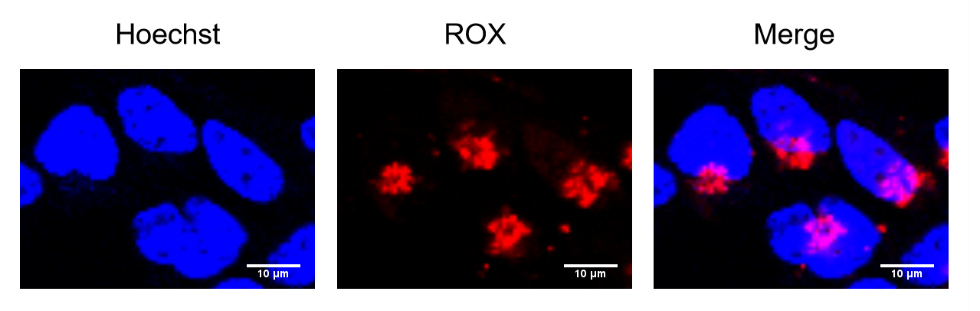


# Figure S5. Imaging of ATED probe (10 nM) in HCT116 cells. The scale bar is 10 μm.
